# Effects of the National Institutes of Health Stroke Scale and Modified Rankin Scale on Predictive Models of 30-Day Nonelective Readmission and Mortality After Ischemic Stroke: Cohort Study

**DOI:** 10.2196/69102

**Published:** 2025-05-09

**Authors:** Mai N Nguyen-Huynh, Janet Alexander, Zheng Zhu, Melissa Meighan, Gabriel Escobar

**Affiliations:** 1 Division of Research, Kaiser Permanente, Pleasanton, CA, United States; 2 Department of Neurology, Kaiser Permanente Walnut Creek Medical Center, 1515 Newell Avenue, Walnut Creek, CA, 94596, United States, 1 925-765-8887; 3 Regional Quality, Accreditation, Regulation & Licensing Department, Kaiser Permanente Foundation Hospitals, Oakland, CA, United States

**Keywords:** ischemic stroke, readmission, predictive modeling, mortality, National Institutes of Health Stroke Scale, NIHSS, modified Rankin scale, mRS

## Abstract

**Background:**

Patients with stroke have high rates of all-cause readmission and case fatality. Limited information is available on how to predict these outcomes.

**Objective:**

We aimed to assess whether adding the initial National Institutes of Health Stroke Scale (NIHSS) score or modified Rankin scale (mRS) score at discharge improved predictive models of 30-day nonelective readmission or 30-day mortality poststroke.

**Methods:**

Using a cohort of patients with ischemic stroke in a large multiethnic integrated health care system from June 15, 2018, to April 29, 2020, we tested 2 predictive models for a composite outcome (30-day nonelective readmission or death). The models were based on administrative data (Length of Stay, Acuity, Charlson Comorbidities, Emergency Department Use score; LACE) as well as a comprehensive model (Transition Support Level; TSL). The models, initial NIHSS score, and mRS scores at discharge, were tested independently and in combination with age and sex. We assessed model performance using the area under the receiver operator characteristic (c-statistic), Nagelkerke pseudo-*R*^2^, and Brier score.

**Results:**

The study cohort included 4843 patients with 5014 stroke hospitalizations. Average age was 71.9 (SD 14) years, 50.6% (2537/5014) were female, and 52.1% (2614/5014) were White. Median initial NIHSS score was 4 (IQR 2-8). There were 538 (10.7%) nonelective readmissions and 150 (3.9%) deaths within 30 days. The logistic models revealed that the best performing models were TSL (c-statistic=0.69) and TSL plus mRS score at discharge (c-statistic=0.69).

**Conclusions:**

We found that neither the initial NIHSS score nor the mRS score at discharge significantly enhanced the predictive ability of the LACE or TSL models. Future efforts at prediction of short-term stroke outcomes will need to incorporate new data elements.

## Introduction

Stroke has a massive impact on patients, their caregivers, and the health system. Approximately 795,000 stroke cases occur annually in the United States. It is the fifth leading cause of death and the leading cause of long-term disability, with nearly 7 million stroke survivors in the United States. With an increasing aging population, it is estimated that nearly 4% of the US adult population would have had a stroke by 2030 [1]. It is all the more tragic that a substantial proportion of stroke survivors will be readmitted to the hospital: current 30-day all-cause readmission rates range from 6.5%‐24.3% and these rates increase to 30%‐62.2% within a year [2]. Mortality following the initial hospitalization is also substantial: the case fatality rate in the hospital is 5%‐7%, increasing to 13%‐15% at 30 days, and 25%‐30% by 1 year [3].

Nearly one-fifth of Medicare beneficiaries discharged from a hospital get readmitted within 30 days [4]. Spurred by a landmark paper by Jencks et al [5] hospitals across the United States have begun implementing strategies to identify and reduce avoidable readmissions. Furthermore, beginning in 2013, 30-day readmission rates for a hospital’s Medicare patients with acute myocardial infarction, heart failure, and pneumonia have been compared with the expected rate of readmissions, using risk adjustment to account for age, gender, medical diagnosis, and selected medical history. Under the Hospital Readmissions Reduction Program, Medicare penalizes hospitals for higher-than-expected rates of readmissions among patients with these diagnoses [6,7]. Additional conditions are likely to be added in the future.

Given the clinical and policy significance of stroke, identifying factors that influence readmission risk is important to assist clinicians and hospitals in the care of patients with stroke and to prevent the preventable. Many factors may contribute to a hospital’s readmission rate after stroke, including patient preferences, demographics, socioeconomic status, comorbidities, stroke severity, health system, clinical care or process, and health outcome. It is important that the risk adjustment mechanisms account for these factors so that hospitals caring for a more complex patient mix are not unduly penalized for readmissions.

The purpose of our investigation was to determine whether we could develop predictive models, specific to patients with stroke, for nonelective readmission or death within 30 days after hospital discharge. In these models, we assessed the ability of commonly used administrative data, newer predictors from the electronic health record (EHR), and 2 manually assigned stroke-specific scales (National Institutes of Health Stroke Scale [NIHSS] and modified Rankin scale [mRS]).

## Methods

### Study Setting

Kaiser Permanente Northern California (KPNC) provides care at 21 Joint Commission certified stroke centers serving 4.5+ million members, who are highly representative of the ethnic and socioeconomic diversity of the surrounding and statewide population [8]. There are approximately 1.3 million ED visits and 3500 ischemic stroke discharges per year. Patients are cared for by a single provider group of 9600 physicians (including approximately 100 neurologists) of The Permanente Medical Group, Inc. In 2016, KPNC implemented the Stroke EXPRESS (Expediting the Process of Evaluating and Stopping Stroke) program [9]. The program included immediate evaluation by a teleneurologist, expedited intravenous thrombolytic treatment, rapid computed tomography angiographic study, and expedited transfer and treatment for patients with large vessel occlusion. Deployment of the Epic EHR [10] was completed in mid-2010.

### Study Cohort

The study cohort included patients meeting the following criteria: (1) admitted to a KPNC hospital between June 15, 2018, and April 29, 2020, (2) at least 18 years of age, (c) diagnosis of incident ischemic stroke (*ICD-10* codes: I63.xx, see Multimedia Appendix 1), and (4) for patients who experienced interhospital transport, the first hospital stay was at a KPNC hospital. Patients with in-hospital deaths were excluded from the final analysis of the study cohort.

The dependent variable for our analyses was a composite outcome (nonelective readmission or death within 30 days of discharge from index event). We defined nonelective readmission as one that began in the emergency department. These were captured from KPNC databases using methods we have recently described [11].

### Data Collection

We obtained the following data elements for all patients directly from the EHR: demographic data; traditional claims (administrative) data (admission and discharge dates, diagnosis and procedure codes, and discharge disposition); newer clinical data such as laboratory test results; vital signs; neurological examinations as recorded in nursing flow sheets; admission and discharge care directives (code status) [12]; bed histories [13]; and length of stay. We assigned all patients a Charlson Comorbidity Index score [14].

As a measure of stroke severity, we captured the initial NIHSS score [15] electronically from the EHR. NIHSS score ranged from 0 to 42, with higher score indicated higher severity and poorer prognosis. Preferences were given to NIHSS performed by neurologists, then by emergency department physicians, then by hospitalists, and finally by nursing. As a measure of functional status at the time of discharge which may influence the patient’s ability to care for self at home and subsequently contribute to readmission, we captured the modified mRS at discharge, which has been mandated for all patients with stroke. The mRS score ranged from 0 to 6, with 0 being no disability and 6 being dead. On a daily basis, the study team identified all patients with stroke admitted in KPNC hospitals. This list was reviewed by 2 research assistants who communicated daily with the local stroke coordinators to ensure that the mRS score has been completed before discharge. If unable to complete before discharge, the research team contacted the patients and their caregivers to complete the equivalent mRS-9Q [16] through phone within 7 days of discharge. The mRS-9Q has been validated for use in person or by telephone.

We also evaluated the following in-hospital data elements as potential predictors: presence of a foley catheter, feeding tube, tracheostomy, peg tube, whether an interpreter was needed, and Functional Independence Measurement (FIM) [17,18] score components. However, the rate of missing data was high without full individual chart reviews (for example, the FIM was only found in 11% of hospitalizations). We did not include these variables in our analyses. Validation of these variables was beyond the scope of our project at this time.

We also captured patients’ longitudinal comorbidity burden and severity of illness at admission. At KPNC, all adults with a medical record number are assigned a Comorbidity Point Score monthly, version 2 (COPS2), which is based on CMS Hierarchical Condition Categories (score range, 0‐1014 [scores above 300 are rare], with higher scores indicating increased mortality risk) [12]. The score is assigned based on all diagnoses a patient has accrued in the preceding 12 months. Patients are also assigned a Laboratory-based Acute Physiology Score, version 2 (LAPS2; range 0‐414 [scores above 200 are uncommon] on admission and every hour after hospitalization, with higher scores indicating worsening instability). The LAPS2 incorporates patients’ vital signs, neurological status checks, pulse oximetry, and 16 laboratory tests. For example, in July 2018, the median hourly LAPS2 among all patients in the intensive care unit was 110, whereas the median ward score was 52. It is not possible to admit a patient to KPNC hospitals without specifying code status, which can be subsequently updated. We classified each patient’s care directive as full code or not (which included partial code, do not resuscitate, and comfort care only).

For our outcomes, we examined readmission within 30 days or mortality within 30 days postdischarge. For readmission and mortality within 30 days, there were no missing data. For those who were readmitted to a non-Kaiser hospital, we identified their readmission through claim data. The study was limited to health plan members only, for whom we had full data on mortality.

Using the above data elements, we assigned all patients a LACE (Length of Stay, Acuity, Charlson Comorbidities, Emergency Department Use) score [19], which is commonly employed in some Kaiser Permanente regions as well as in other hospital systems. The LACE is widely used to predict a 30-day readmission or mortality composite outcome. We also assigned patients a Transition Support Level (TSL) score [20]. The TSL, an automated score that was developed in KPNC included the LAPS2, COPS2, recent use, and a patient’s care directive on the day of discharge, was assigned in real time daily to all KPNC hospitalized adults.

The paper adheres to recommended reporting guidelines for observational studies, and the STROBE (Strengthening the Reporting of Observational Studies in Epidemiology) guidelines (Checklist 1) were followed.

### Statistical Analysis

We performed bivariate comparisons using chi-square and *t* tests. We fit logistic regression models to predict the composite outcome. We tested the following as independent predictors: NIHSS, mRS, LACE, and TSL; the individual components of the LACE and TSL; and combinations of these (NIHSS+age+sex; MRS+age+set; LACE+NIHSS, LACE+MRS; TSL+NIHSS, TSL+MRS). To establish the probable maximum performance of these predictors, we also tested a random forest model that included age, sex, NIHSS, mRS, and all of the individual predictors included in the LACE and TSL.

We evaluated model performance using the area under the receiver operator characteristic curve (c statistic, the Nagelkerke pseudo-*R*^2^, and the Brier score) [21-23]. All models were assessed using 5-fold cross validation, and the models’ performance metrics are those based on 5-fold cross validation. All analyses were done in SAS (version 9.3; SAS Institute).

### Ethical Considerations

All KPNC facilities are under the jurisdiction of one institutional review board for the protection of human participants. This study was approved by the KPNC Institutional Review Board, which waived the requirement for written informed consent (IRB number: 1279378). Only aggregate data is provided here. Participants received no compenstation from the study.

## Results

Between June 15, 2018, and April 29, 2020, there were 5808 hospitalizations for adult patients with ischemic stroke. Of these 5808 hospitalizations, 794 (13.7%) were excluded from the study analysis because the patient died during the index hospitalization (n=198, 3.4%), the hospitalization originated at a non-KPNC facility (n=504, 8.7%), or follow-up data was incomplete (n=92, 1.6%). The final study population included 5014 hospitalizations. The average age of the study population was 71.9 (SD 13.99) years and 49.4% (2477/5014) of patients were male. The racial and ethnic distribution was 52.1% (2614/5014) White, 15.6% (782/5014) Hispanic, 15% (751/5014) Asian, and 14.3% (718/5014) Black. The median initial NIHSS score was 4 (IQR 2-8). The average mRS score at discharge was 2.8 (SD 1.64). Mean length of stay was 6.5 days (SD 7.71), and 55% (2756/5014) were discharged home. Table 1 presents the other characteristics of the study population.

**Figure 1.**
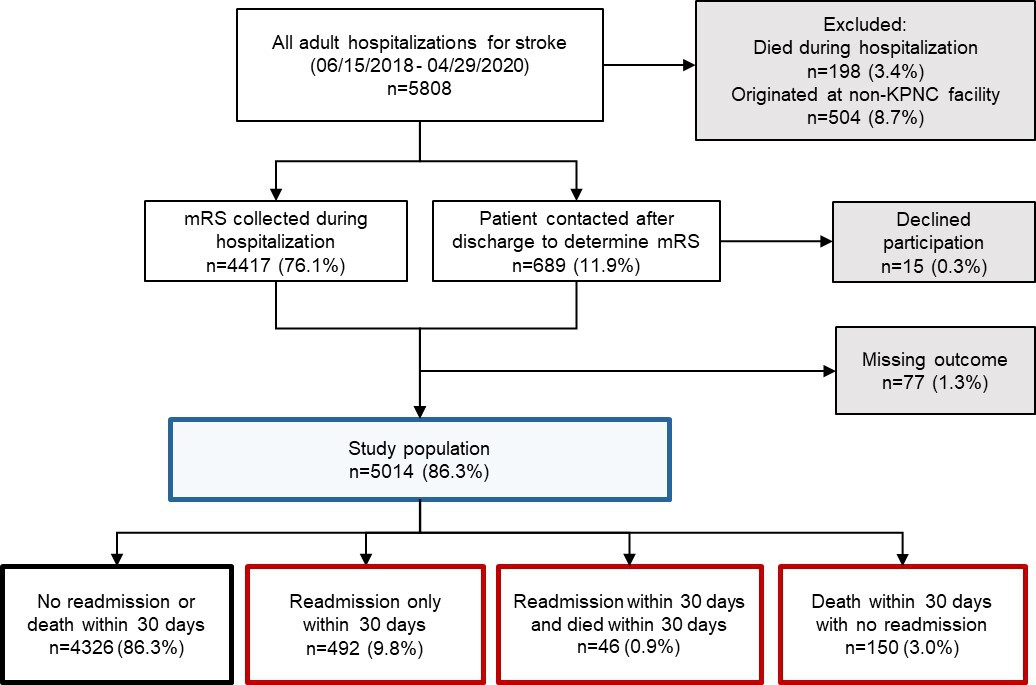
Definition of study cohort. Eligible patients are those patients meeting the following criteria: (1) admitted to a KPNC hospital between June 15, 2018, and April 29, 2020; (2) age ≥18 years; (3) diagnosis of incident ischemic stroke; and (4) for patients who experience interhospital transport, the first hospital stay was at a KPNC hospital. KPNC: Kaiser Permanente Northern California; mRS: modified Rankin Scale.

Out of 5014 hospitalizations, 688 (13.7%) had a nonelective readmission within 30 days or died within 30 days postdischarge (composite outcome). Out of 5014 hospitalizations, 492 (9.8%) had a readmission only within 30 days, 150 (3.0%) died with no readmission within 30 days, and 46 (0.9%) had both a readmission within 30 days and died within 30 days (see Figure 1 and Table 1). Patients who died but had no readmission within 30 days, had an elevated history of atrial fibrillation (47/150, 31.3%). Patients who had no readmission or death within 30 days tended to be more likely to have received alteplase during the index hospital stay (639/4326, 14.8%). The proportion of patients with full code orders at discharge varied across patient groups (range 8.7%-80.4%). The proportion of patients who were discharged home after the index event varied by outcome group. Of those who were both readmitted and died, 28% (13/46) had been released to home, compared with 40.7% (200/492) of those who were readmitted and alive, and 56.2% (2433/4326) of those who were alive and had no readmission. In total, 50% of patients (23/46) who died within 30 days but had no readmission within 30 days, had been released to a regular (noncustodial) skilled nursing facility. Furthermore, 4% (6/150) of patients with death but no readmission, were originally discharged to a custodial skilled nursing facility (Table 1).

For hospitalizations that resulted in readmission within 30 days or death within 30 days post-discharge, the patients tended to be older (median age 78, IQR 68-86 years vs 72, IQR 62-82 years), were less likely to be male (290/688, 42.2% vs 2187/4326, 50.6%; *P*<.001), and more likely to have a Charlson comorbidity score greater than or equal to 4 (387/688, 56.3% vs 1584/4326, 36.6%; *P*<.001). More patients without the composite outcome had full code care directive at discharge (3476/4326, 80.4% vs 402/688, 58.4%; *P*<.001; see Table 2).

Table 3 shows results from performance testing of several multivariable predictive models of the composite outcome. The best performing models were TSL (c-statistic=0.685) and TSL plus mRS score at discharge (c-statistic=0.694). The model with TSL+NIHSS performed similarly (c-statistic=0.684) to the model with TSL alone (Table 3).

**Table 1.** Cohort characteristics. Study composite outcome is nonelective readmission and/or death within 30 days of discharge.

Variable	No readmission or death within 30 days	Readmission only within 30 days	Readmission within 30 days and died within 30 days	Death within 30 days only	Composite outcome	All patients
Hospitalizations, n	4326	492	46	150	688	5014
All patients, n	4250	481	46	150	669	4843
Age (years), mean (SD)	71.2 (13.99)	73.9 (13.08)	78.4 (11.63)	83.2 (11.48)	76.2 (13.21)	71.9 (13.99)
Male, n (%)	2187 (50.6)	219 (44.5)	21 (45.7)	50 (33.3)	290 (42.2)	2477 (49.4)
Race and ethnicity, n (%)						
Asian	654 (15.1)	74 (15)	5 (10.9)	18 (12)	97 (14.1)	751 (15)
Black	620 (14.3)	77 (15.7)	6 (13)	15 (10)	98 (14.2)	718 (14.3)
Hispanic	674 (15.6)	79 (16.1)	6 (13)	23 (15.3)	108 (15.7)	782 (15.6)
Other or multiracial	134 (3.1)	10 (2.0)	1 (2.2)	4 (2.7)	15 (2.2)	149 (3.0)
White	2244 (51.9)	252 (51.2)	28 (60.9)	90 (60)	370 (53.8)	2614 (52.1)
KFHP^a^ membership, n (%)	3634 (84)	452 (91.9)	44 (95.7)	135 (90)	631 (91.7)	4265 (85.1)
Inpatient incident stroke, n (%)	397 (9.2)	38 (7.7)	1 (2.2)	14 (9.3)	53 (7.7)	450 (9)
Medical history, n (%)						
Previous stroke	199 (4.6)	34 (6.9)	3 (6.5)	10 (6.7)	47 (6.8)	247 (4.9)
Atrial fibrillation	636 (14.7)	96 (19.5)	8 (17.4)	47 (31.3)	151 (21.9)	789 (15.5)
Myocardial infarction	109 (2.5)	24 (4.9)	3 (6.5)	4 (2.7)	31 (4.5)	140 (2.7)
COPS2^b^, mean (SD)	35.4 (35.74)	49.4 (43.95)	69.8 (52.12)	64.6 (47.71)	54.1 (45.92)	38.0 (37.85)
≥65^c^, n (%)	755 (17.5)	141 (28.7)	22 (47.8)	68 (45.3)	231 (33.6)	986 (19.7)
LAPS2^d^ at admission, mean (SD)	58.1 (29.63)	69.0 (33.05)	83.9 (24.81)	87.0 (30.95)	74.0 (33.01)	60.3 (30.60)
≥110^e^, n (%)	252 (5.8)	61 (12.4)	7 (15.2)	37 (24.7)	105 (15.3)	357 (7.1)
Received alteplase, n (%)	639 (14.8)	52 (10.6)	4 (8.7)	13 (8.7)	69 (10)	708 (14.1)
mRS^f^ score at discharge, mean (SD)	2.7 (1.63)	3.1 (1.57)	3.7 (1.37)	3.7 (1.78)	3.3 (1.62)	2.8 (1.64)
Initial NIHSS^g^ score, median (IQR)	4 (2-8)	4 (2-9)	5 (3-8)	15 (6-22)	6 (2-13)	4 (2-8)
Full code at discharge, n (%)	3476 (80.4)	366 (74.4)	23 (50)	13 (8.7)	402 (58.4)	3878 (77.3)
LOS^h^ in days, mean (SD)	6.4 (7.91)	6.7 (6.37)	7.7 (7.19)	7.3 (5.74)	6.9 (6.30)	6.5 (7.71)
Discharge disposition, n (%)						
CSNF^i^	13 (0.3)	0 (0)	0 (0)	6 (4)	6 (0.9)	19 (0.4)
Home health	1067 (24.7)	121 (24.6)	10 (21.7)	8 (5.3)	139 (20.2)	1206 (24.1)
Home	2433 (56.2)	200 (40.7)	13 (28.3)	110 (73.3)	323 (46.9)	2756 (55)
RSNF^j^	813 (18.8)	171 (34.8)	23 (50)	26 (17.3)	220 (32)	1033 (20.6)

a KFHP: Kaiser Foundation Health Plan.

b COPS2: Comorbidity Point Score, version 2.

c COPS2 (range: 0 to 1010; higher scores indicate increasing comorbidity burden) is assigned based on all diagnoses incurred by a patient in the 12 months preceding the index hospitalization. The univariate relationship of COPS2 with 30-day mortality is as follows: 0-39, 1.7%; 40-64, 5.2%; 65+,9.0%. See Escobar et al [12] for details.

d LAPS2: Laboratory-based Acute Physiology Score, version 2.

e LAPS2 (range: 0 to 414; higher scores indicating increasing physiologic derangement) is assigned based on a patient’s worst vital signs, pulseoximetry, neurological status, and 16 laboratory test results in the 72 hours preceding hospitalization. The univariate relationship of an admission LAPS2 with 30-day mortality is as follows: 0-59, 1.0%; 60-109, 5.0%; 110+, 13.7%. See text and Escobar et al [12] for details.

f mRS: modified Rankin Scale.

g NIHSS: National Institutes of Health Stroke Scale.

h LOS: length of stay.

i CSNF: custodial skilled nursing facility.

j RSNF: regular skilled nursing facility.

**Table 2.** Distribution of key predictors in the study cohort.

	Patients without study composite outcome	Patients with study composite outcome	*P* value
Age (years), median (IQR)	72 (6282)	78 (68-86)	—^a^
Sex (male), n (%)	2187 (50.6)	290 (42.2)	<.001
Admitted via ED^b^, n (%)	3929 (90.8)	635 (92.3)	.21
CCI^c^ score, median (IQR)^d^	2 (0-5)	4 (2-6)	—
CCI ≥4, n (%)	1584 (36.6)	387 (56.3)	<.001
Length of stay, median (IQR)^e^	4 (3-6)	5 (3-6)	—
mRS^f^ score, median (IQR)^g^	3 (1-4)	4 (2-4)	—
mRS >2, n (%)	2119 (60.1)	425 (73.9)	<.001
NIHSS^h^ score, median (IQR)^i^	4 (2-7)	5 (2-13)	—
NIHSS >5, n (%)	1270 (35)	272 (50.1)	<.001
Full code at discharge, n (%)^j^	3476 (80.4)	402 (58.4)	<.001
LACE^o^, median (IQR)^l^	7 (4-10)	9 (6-12)	—
COPS2^m^, median (IQR)^n^	21 (10-50)	42 (13-81.5)	—
LAPS2^k^, median (IQR)^p^	55 (36-75)	72.5 (50-94)	—
Prior hospitalizations, n (%)^q^	168 (3.9)	56 (8.1)	<.001
TSL^r^ score, median (IQR)^s^	10.7 (7.5-17.6)	18.2 (10.7-29.1)	—

a Not available.

b ED: emergency department.

c CCI: Charlson Comorbidity Index.

d CCI score (range 0-40; higher scores indicate greater comorbidity burden) was calculated using the methodology of Deyo et al [14].

e Length of stay is extracted in hours and reported in days, rounded to the nearest 0.1 day.

f mRS: modified Rankin Scale.

g mRS score range is 0-6 (0=no symptoms at all,1=no significant disability despite symptoms, 2=slight disability, 3=moderate disability, 4=moderately severe disability 5=severe disability/bedridden, 6=dead). See text and van Swieten et al [24] for details.

h NIHSS: National Institutes of Health Stroke Scale.

i NIHSS score range is 0-42 (0-5 typically indicates minor stroke; higher scores indicate more severe stroke). See text and Brott et al [25] for details.

j Assignment is based on patient’s last care directive before discharge.

k LACE: Length of Stay, Acuity, Charlson Comorbidities, Emergency Department Use.

l LACE score range is 0 to 19, with higher scores indicating increased risk of the composite outcome. See text and van Walraven et al [19] for details.

m COPS2: Comorbidity Point Score, version 2.

n COPS2 (range: 0 to 1010; higher scores indicate increasing comorbidity burden) is assigned based on all diagnoses incurred by a patient in the 12 months preceding the index hospitalization. The univariate relationship of COPS2 with 30-day mortality is as follows: 0-39, 1.7%; 40-64, 5.2%; 65+, 9.0%. See Escobar et al [12] for details.

o LAPS2: Laboratory-based Acute Physiology Score, version 2.

p LAPS2 (range: 0 to 414; higher scores indicating increasing physiologic derangement) is assigned based on a patient’s worst vital signs, pulse oximetry, neurological status, and 16 laboratory test results in the 72 hours preceding hospitalization. The univariate relationship of an admission LAPS2 with 30-day mortality is as follows: 0-59, 1.0%; 60-109, 5.0%; 110+, 13.7%. See text and Escobar et al [12] for details.

q Number with any hospitalizations in the 30 days preceding the index hospitalization.

r TSL: transition support level.

s This is expressed as a % risk of the composite outcome within 30 days of hospital discharge. The score incorporates the LAPS2, COPS2, discharge care directive, and previous hospitalizations in the 30 days preceding the index hospitalization. Patients with TSL scores ≥25% are enrolled in Kaiser Permanente Northern California readmission prevention program. See text and Escobar et al [20] for details.

**Table 3.** Multivariate model performance^a^.

Model	c-statistic	Nagelkerke pseudo-*R*^2^	Brier score
NIHSS^b^	0.60	0.03	0.11
NIHSS+age+sex	0.64	0.05	0.11
mRS^c^	0.61	0.03	0.12
mRS+age+sex	0.62	0.04	0.12
LACE^d^	0.63	0.04	0.12
LACE+NIHSS	0.66	0.06	0.11
LACE+mRS	0.67	0.07	0.12
TSL^e^	0.69	0.08	0.11
TSL+NIHSS	0.68	0.09	0.11
TSL+mRS	0.69	0.10	0.11
Random forest^f^	0.66	0.04	0.11

a All models except the random forest model employed logistic regression. Metrics reported: c-statistic (area under the receiver operator characteristic curve); Nagelkerke pseudo-R2 (Nagelkerke [22] and Brier score [23].

b NIHSS: National Institutes of Health Stroke Score (range 0-42; higher scores indicate more severe stroke). See text and Brott et al [25] for details.

c mRS: modified Rankin Scale, (range 0-6; higher scores indicate severe disability or death). See text and van Swieten et al [24] for details.

d LACE: Length of Stay, Acuity, Charlson Comorbidities, Emergency Department Use. Score range is 0 to 19, with higher scores indicating increased risk of the composite outcome. See text and van Walraven et al [19] for details.

e TSL: Transition Support Level. This score is expressed as a % risk of the composite outcome within 30 days of hospital discharge. The score incorporates the Laboratory-based Acute Physiology Score, version 2; Comorbidity Point Score, version 2; discharge care directive; and previous hospitalizations in the 30 days preceding the index hospitalization. Patients with TSL scores ≥25% are enrolled in Kaiser Permanente Northern California readmission prevention program. See text and Escobar et al [20] for details.

f For the random forest model, we included age, sex, NIHSS, mRS, and all subcomponents of the LACE and TSL scores.

## Discussion

We have completed a study where we developed an adult patient stroke-specific predictive model for a composite outcome of readmission within 30 days and mortality within 30 days postdischarge. Among the models that were tested, the TSL model performed the best. Adding manually assigned stroke scales such as initial NIHSS (as a measure of stroke severity) and mRS at discharge (as a measure of functional status) to clinical data from a comprehensive EHR did not improve the performance of the TSL model significantly. The previously published TSL model [20] included an extensive amount of data for each patient including the LAPS2, COPS2, discharge care directive, and previous hospitalizations in the 30 days preceding the index hospitalization. Some of these data (such diagnoses in the previous 12 months before hospitalization, vital signs, laboratory data, and code status) were unique to our dataset and were never examined in previously published studies on 30-day readmission or mortality poststroke.

Readmissions after stroke are associated with worse disability, higher mortality, and higher costs of care [26,27]. Reduction of 30-day readmission is a quality metric set by the Centers for Medicare & Medicaid Services. Publications on readmission have been more focused on reported global performance and other conditions, and less so on stroke. In addition, most previous studies excluded patients aged <65 years. Being able to predict readmission after stroke may help to identify high-risk patients for targeted interventions and postdischarge programs, limit preventable readmissions, and improve long-term outcomes.

Previously published studies on readmissions after stroke provided only limited data on patient-level factors in their risk analysis. A systematic review of stroke readmission in 2010 identified only 16 published studies that reported risk-adjustment models at the patient level [2]. Considerable variation in reported outcomes was identified: 30-day all-cause readmission rates ranged from 6.5% to 24.3%, 1-year all-cause readmission rates from 30% to 62.2%, 30-day stroke-related readmission from 7.4% to 9.4%, and 1-year stroke-related readmission from 10.5% to 31.1%. Patient characteristics that were found to be associated with stroke readmissions included age, longer index hospital length of stay, worse physical functioning after stroke, and increased number of hospitalizations before stroke. Although some of these included stroke severity measures, no consistency existed across the models. A statement from the American Heart Association on risk adjustment of ischemic stroke outcomes for comparing hospital performance emphasized a minimum list of patient-level variables including age, sex, stroke severity, comorbid conditions, and risk factors [3]. However, in order to improve the usability of risk-adjusted models, the statement recommended that researchers should try to identify other factors, especially those not typically measured in administrative databases. To our knowledge, no previously published models on 30-day readmission or 30-day mortality included the mRS at discharge (a functional status assessment).

A number of studies on readmission and mortality poststroke used Medicare database therefore restricting their cohorts by older age and limited the generalizability of their results [28,29]. Our 30-day readmission rate of 9.7% was lower than rates (12%‐14%) reported from those studies using Medicare data. However, advanced age is associated with increased readmission after ischemic stroke [29]. On the other hand, a recent analysis of 30-day readmission after ischemic stroke for adults 18 years and older using the 2019 Nationwide Readmission Database reported a rate of 9.7%, which was nearly identical to ours [30].

For our cohort, the rate for 30-day mortality postdischarge was 3%, and the inpatient mortality rate was 3.4%, totaling 6.4% for 30 days from admission. Studies from Canada and the United Kingdom reported a 30-day mortality rate from admission of about 12% [31,32]. A US study using Medicare data reported a 30-day mortality rate of 13.6% [33]. Our lower 30-day mortality rate may be a reflection of a cohort of patients with insurance and access to care, inclusion of younger patients (ages 18 years and older), and an excellent performance of acute stroke treatment time as previously published [9].

The current state of predictive analytics for hospital readmission has not evolved enough to guide effective prevention efforts [4,5,34,35]. In addition, medical record technology, along with other aspects of health care in the United States is both fragmented and—from an informatics perspective—heterogeneous. Some institutions, such as university medical centers and KPNC, have sophisticated EHRs, while others still use paper charts. Similarly, the ability of institutions to retrieve electronic data varies considerably, as does the timeliness of such retrieval. Given this heterogeneous information environment, it is important to (1) highlight desirable characteristics of future readmission predictive models for patients with stroke, and (2) develop models that can be instantiated in different settings (ie, ranging from those using only claims data to those that use comprehensive EHRs). This would permit the development of modular approaches that individual institutions could adapt to their specific circumstances.

It was not surprising that models using only administrative data (LACE) did not perform well. However, it was disappointing that the TSL model with inclusion of detailed EHR data (longitudinal comorbidity in the 12 months preceding index hospitalization, COPS2; and LAPS2) did not result in great discrimination. Adding the initial NIHSS score and the mRS score at discharge to the model did not improve its performance, perhaps because the TSL model at baseline already contained other data elements relating to patient status and severity of their condition while hospitalized. NIHSS and mRS were moderately correlated to LAPS2 score, LACE, and TSL. We did not have other potential measures of stroke severity such as infarct size from neuroimaging study, or change in mRS at discharge compared with baseline prehospital mRS, or mRS postdischarge.

Our study had several strengths. Our approach synthesizes two types of expertise: deep understanding of the clinical aspects of stroke (including intimate familiarity with the current state of the art in assessment of its severity and prognosis) combined with sophistication in the area of predictive analytics. Tools developed by our experienced predictive analytics team have entered routine operations in one of the most advanced integrated health care delivery systems in the United States. We were able to characterize the study cohort better than has been done previously. The TSL model included detailed data from index hospitalization not in previous studies on readmission poststroke, including vital signs, laboratory data, patient’s care directive and mRS score at discharge. This study was embedded in a community setting with comprehensive longitudinal information infrastructure. The study cohort was diverse in race-ethnicity and included all ages, not just Medicare-eligible patients. We used prospective validation. All analyses were based on two basic data standards: claims data (functionally, the lowest common denominator) and modern EHR data. This study started from the premise that prediction of a patient with stroke‘s risk for readmission should be automated, presented through a comprehensive EHR, and occur in real time.

There were also limitations with our study. We relied on data collected in the course of routine clinical operations rather than strict research protocols. While our hospitals treat patients with stroke regardless of insurance, our study cohort and analysis were limited to an insured population with access to care to one of the most highly integrated health care delivery systems in the world. The study results may not be applicable to the uninsured population. All of our centers are Joint Commission certified stroke centers with the highest awards from the Get With the Guidelines program. More than 50% of acute ischemic stroke cases qualifying for intravenous thrombolytic were treated within 30 minutes [9]. Therefore, our study results may not be representative of all hospitals caring for patients with ischemic stroke. We did not have baseline functional status and, therefore, could not include a change in functional status to the models. We also did not have indicators for postdischarge functional status. We were unable to include other in-hospital data such as placement of feeding tube or trach due to a high rate of missing data perhaps due to difficulty with pulling correct data electronically. We did not have data on social support or a cognitive function measure at discharge, which may play a significant role in readmission. Our study results are generalizable to an adult population with ischemic stroke who have insurance and access to care who survive the index hospitalization and are treated in certified stroke centers.

In summary, we have tested a more comprehensive predictive model (TSL) for readmission and death within 30 days post ischemic stroke. Having a comprehensive look at the important factors that may influence that decision-making process (socioeconomic status, living arrangement or social support, functional status, code status, cognitive status, etc) would go a long way at improving the performance of a predictive model. Future studies should seek to improve upon our study and evaluate other factors not included here, such as changes from baseline functional status, the volume of brain infarcted, social support, and cognitive status at the time of discharge.

## Supplementary material

10.2196/69102 Multimedia Appendix 1 *ICD-10* codes for diagnosis of ischemic stroke. *ICD-10*: *International Classification of Diseases, Tenth Revision*.

10.2196/69102 Checklist 1 STROBE (Strengthening the Reporting of Observational Studies in Epidemiology) checklist.

## Abbreviations

CCI: Charlson Comorbidity Index
COPS2: Comorbidity Point Score, version 2
CSNF: custodial skilled nursing facility
ED: emergency department
EHR: electronic health record
EXPRESS: Expediting the Process of Evaluating and Stopping Stroke
FIM: Functional Independence Measurement
KFHP: Kaiser Foundation Health Plan
KPNC: Kaiser Permanente Northern California
LACE: Length of Stay, Acuity, Charlson Comorbidities, Emergency Department Use
LAPS2: Laboratory-based Acute Physiology Score, version 2
mRS: modified Rankin Scale
NIHSS: National Institutes of Health Stroke Scale
RSNF: regular skilled nursing facility
STROBE: Strengthening the Reporting of Observational Studies in Epidemiology
TSL: Transition Support Level
