# Supplementary material for: Effects of the National Institutes of Health Stroke Scale and Modified Rankin Scale on Predictive Models of 30-Day Nonelective Readmission and Mortality After Ischemic Stroke: Cohort Study
Source: JMIR Med Inform. 2025 May 9;13:e69102. doi: 10.2196/69102 (PMC12083732; doi:10.2196/69102)
Supplement: Multimedia Appendix 1 [file medinform-v13-e69102-s001.docx]

**Appendix**

ICD-10 codes for cohort identification: I63.02, I63.12, I63.22, I63.031, I63.032, I63.039, I63.131, I63.132, I63.139, I63.231, I63.232, I63.239, I63.011, I63.012, I63.019, I63.111, I63.112, I63.119, I63.211, I63.212, I63.219, I63.59, I63.09, I63.19, I63.59, I63.00, I63.10, I63.20, I63.29, I63.30, I63.311, I63.312, I63.319, I63.321, I63.322, I63.329, I63.331, I63.332, I63.339, I63.341, I63.342, I63.349, I63.39, I63.6, I63.40, I63.411, I63.412, I63.419, I63.421, I63.422, I63.429, I63.431, I63.432, I63.439, I63.441, I63.442, I63.449, I63.49, I63.50, I63.511, I63.512, I63.519, I63.521, I63.522, I63.529, I63.531, I63.532, I63.539, I63.541, I63.542, I63.549, I63.59, I63.8, I63.9
